# Insurance Customers’ Expectations for Sharing Health Data: Qualitative Survey Study

**DOI:** 10.2196/16102

**Published:** 2020-03-26

**Authors:** Casandra Grundstrom, Olli Korhonen, Karin Väyrynen, Minna Isomursu

**Affiliations:** 1 Faculty of Information Technology and Electrical Engineering University of Oulu Oulu Finland

**Keywords:** data sharing, qualitative research, survey, health insurance, insurance, medical informatics, health services

## Abstract

**Background:**

Insurance organizations are essential stakeholders in health care ecosystems. For addressing future health care needs, insurance companies require access to health data to deliver preventative and proactive digital health services to customers. However, extant research is limited in examining the conditions that incentivize health data sharing.

**Objective:**

This study aimed to (1) identify the expectations of insurance customers when sharing health data, (2) determine the perceived intrinsic value of health data, and (3) explore the conditions that aid in incentivizing health data sharing in the relationship between an insurance organization and its customer.

**Methods:**

A Web-based survey was distributed to randomly selected customers from a Finnish insurance organization through email. A single open-text answer was used for a qualitative data analysis through inductive coding, followed by a thematic analysis. Furthermore, the 4 constructs of commitment, power, reciprocity, and trust from the social exchange theory (SET) were applied as a framework.

**Results:**

From the 5000 customers invited to participate, we received 452 surveys (response rate: 9.0%). Customer characteristics were found to reflect customer demographics. Of the 452 surveys, 48 (10.6%) open-text responses were skipped by the customer, 57 (12.6%) customers had no expectations from sharing health data, and 44 (9.7%) customers preferred to abstain from a data sharing relationship. Using the SET framework, we found that customers expected different conditions to be fulfilled by their insurance provider based on the commitment, power, reciprocity, and trust constructs. Of the 452 customers who completed the surveys, 64 (14.2%) customers required that the insurance organization meets their data treatment expectations (commitment). Overall, 4.9% (22/452) of customers were concerned about their health data being used against them to profile their health, to increase insurance prices, or to deny health insurance claims (power). A total of 28.5% (129/452) of customers expected some form of benefit, such as personalized digital health services, and 29.9% (135/452) of customers expected finance-related compensation (reciprocity). Furthermore, 7.5% (34/452) of customers expected some form of empathy from the insurance organization through enhanced transparency or an emotional connection (trust).

**Conclusions:**

To aid in the design and development of digital health services, insurance organizations need to address the customers’ expectations when sharing their health data. We established the expectations of customers in the social exchange of health data and explored the perceived values of data as intangible goods. Actions by the insurance organization should aim to increase trust through a culture of transparency, commitment to treat health data in a prescribed manner, provide reciprocal benefits through digital health services that customers deem valuable, and assuage fears of health data being used to prevent providing insurance coverage or increase costs.

## Introduction

### Background

The paradigm shift toward person-centric health care promotes the engagement of individuals in their care process, providing pervasive digital interventions for preventative and proactive health and wellness [1,2]. At the core of this paradigm shift are health data, as data usage is the cornerstone for health care and pivotal for the success of the health data economy [3]. Individuals are being empowered with health data sharing capabilities through mechanisms such as interoperability and portability, which are slated to facilitate a wide range of financial, academic, societal, and personal benefits for health and wellness [4]. For example, patient-led health data sharing on the digital platform, *PatientsLikeMe*, has led to better outcomes in areas such as symptom management and medication adherence [5]. Similarly, monitoring platforms for home-based self-measurements can support the decision-making processes of health care professionals using real-time patient-generated health data [6]. The mechanisms for health data sharing are enacted in the General Data Protection Regulation (GDPR) throughout Europe and implicate data processors and controllers to facilitate desirable outcomes such as access to health data [7]. Consequently, traditional business models are being disrupted and transformed to sustain organizations, now that the power to control health data has been shifted toward data subjects such as patients, individuals, or customers [8-11].

Extant literature has mostly focused on the barriers of health data sharing in the public setting [4], emphasizing personalization, care improvement, and the intended use of health data as conditions that facilitate sharing [12-14]. Objections to health data sharing with insurance organizations are much more prevalent in the existing literature. Nearly half of the participants in a study in the United States, including doctors and patients, were found to strongly object to disclosing health data because of concerns about discrimination. The participants expected their shared health data to be used as leverage to impede medical care or insurance coverage [15]. In the Nordic countries especially, there is only limited empirical research on customers sharing health data in the private insurance sector [16]. In particular, there is a lack of case study research wherein relevant stakeholders are studied holistically to develop insights into the motivations for and perceptions about sharing health data [17]. Studies that are available indicate similar findings; the majority of individuals are unwilling to share their health data with insurance organizations. Approximately 57% of customers from a Finnish insurance organization indicated an unwillingness to share their health data [18]. Further conflating the matter, institutional barriers within insurance organizations can obstruct obtaining a holistic understanding of the customers’ willingness to share health data [19]. A study in Canada reported that an overwhelming 79% of physicians and 67% of patients do not want private insurance organizations to have access to health data, even if anonymized or used for research [20], depicting private insurance organizations to be more untrustworthy than the pharmaceutical industry or the government for sharing health data.

Severe distrust is reflected across other health data sharing studies as well, especially when insurance organizations are suspected of profiting from selling or using health data—an unintended consequence of health data sharing [21-23]. When customers perceive that organizations are only using their data for self-interest value creation, customers will not be motivated to share their data and ultimately lose trust in the organization [24]. The social exchange theory (SET) presumes that to get something, you must give something of equivalent perceived value in return [25]. In the context of the insurance industry, this would mean that for customers to share their health data, the insurance organization would need to offer the customers something in return. What this *something* is, however, is unclear.

### Objectives

As previous research indicates, there exists a high reluctance of individuals to share health data across the insurance industry. However, at the same time, a need for health data sharing to drive digital health services is increasing as part of the data economy, marking a clear research gap. In response to this gap, we asked the following question: What would customers expect in return for sharing their health data?

We aimed to address this gap by investigating the expectations of insurance customers for sharing their health data. Furthermore, we sought to garner insight into the customers’ perceived values that are intrinsic to their expectations when sharing health data. Finally, we explored the conditions that the insurance organizations should facilitate to incentivize customers’ health data sharing, all to aid in the design and development of proactive digital health services.

## Methods

### Case Study

The insurance organization chosen for this case study is one of the largest in Finland. For anonymity purposes, the moniker *Omega* is used. In Finland, health care is mostly decentralized and has three main avenues of distribution: primary public health care; occupational health care, which all employers are obligated to provide; and private services, where voluntary private insurance exists [26]. Omega offers both occupational health care and private health care services, including a Web-based virtual hospital. As business models are transforming in response to an abundance of data [8], Omega is currently making strategic movements away from traditional insurance models to a more proactive one. To be able to provide more proactive health services that help prevent illness or injury to the customer, Omega requires access to customers’ health data [27,28]. The control of how any type of data is shared or managed affects the organizations that rely on it. Understanding the conditions under which customers will share data is vital for driving the shift in preventative health care provision. This change makes Omega highly suitable for this study to determine customers’ expectations in sharing health data.

### Survey Design and Development

The information provided about the survey follows the *Checklist for Reporting Results of Internet E-Surveys* [29]. The survey was designed iteratively. It included a variety of survey utilities that were both quantitative and qualitative in nature. No randomization of question order was included. There were 5 screens (ie, 4 subject areas and informed consent) and a total of 23 questions across 4 key subject areas: customer characteristics (5 questions), health data (7 questions), value creation (6 questions), and social media (5 questions). The survey was pilot tested with 4 Finnish testers through Web-based testing and feedback and 2 cognitive walkthroughs in English and Swedish. The feedback was summarized and used to iterate the next version of the survey. The content and clarity of questions were improved to ensure uniform meaning across the 3 languages (ie, Finnish, Swedish, and English) in which the survey was available. Native speakers were involved when translating the survey into all 3 languages. A check for completeness was added, as were nonresponse options, the ability to review answers by moving back and forth through the survey, and a progress bar to indicate the percentage of completion at the bottom of the screen.

### Data Collection

A probability-based sampling technique was used within the population of Omega’s customers as the response rate in previously distributed surveys varied based on topic and was therefore unpredictable. A simple random sampling approach was chosen to provide a high degree of sample probability and minimize probabilistic sampling method biases [30]. The survey itself was randomly allocated to 5000 customers with the only constraints being that respondents had to be Omega’s existing customers aged older than 18 years (for consent purposes) and had to have email addresses (so they may be reached). A minimum of 385 responses from Omega’s customer base of approximately 1.3 million was required to accommodate our goal of acquiring a 95% CI (descriptive statistics of the same survey [18]). A total of 452 viable responses were received from 5000 customers (response rate: 9.0%) selected as a predetermined sample size. The 452 responses provided us with a rich dataset for a qualitative analysis of free-text answers. In this paper, we did not use the data for the quantitative analysis. The survey was sent on January 30, 2018, and was live for 6 weeks (until March 15, 2018). The time frame was determined by monitoring the number of responses received. Most of the responses were received within the first 15 days.

### Data Analysis

For the purposes of this paper, only the customers’ characteristics and qualitative health data responses have been discussed. More detailed descriptive statistics about Omega can be found in another paper [18]. A single open-text question from the health data section was analyzed. The question was *“*If you were to share your health data with Omega, what would you expect in return?” The *expectation* of the customer was asked in a neutral manner so that the customer was not biased toward negative or positive expectations. The output of the text was intended to be analyzed qualitatively to avoid the pitfall of quasi-content of free text in surveys [31]. A total of 452 translated quotes were extracted from the survey and sequenced into a Microsoft Excel spreadsheet. With the exception of one response, which was in Swedish, all open-text answers were in Finnish and translated to English for analysis. During the analysis, a native Finnish speaker (second author) actively participated to allow for nuances and social contexts to be discussed. Each quote was inductively coded by the first and second authors. No text recognition or automated frequency software was used; all analyses were performed manually to ensure coding familiarity. Once the codes were saturated, similar open-text answers were grouped together through content-driven analysis and then built into themes. Finally, the SET framework was applied (detailed in the following section).

### Social Exchange Theory Framework

With classic origins, the SET is a prominent concept used across a variety of disciplines [32] and has been used in more modern information and digital settings, such as knowledge-sharing power plays in interdisciplinary collaborations [33]. Here, we applied Emerson’s [25] definition of SET, which is built on the work by Blau in sociology [34] and Homans in social psychology [35]. Emerson [25] advocates that SET is in fact not a theory but rather a framework to gather relevant assumptions in the context of structural functionalism where exchange occurs upon the contingency of perceived values. Other disciplines have previously borrowed SET to generate new theoretical insights [35,36]. However, few studies have connected intangible values such as data with social exchanges between organizations and their customers [37]. The SET framework used in our analysis was adopted from a study by Wu et al [38], which synthesized SET issues to develop a model for partner prerequisites for information sharing in supply chains. They determined that for partners to be willing to share information, the constructs of commitment, power, reciprocity, and trust need to be established. This framework was suitable for adoption into our context as data are entangled with supply and value chains in organizations. More specifically, in the case of an insurance organization strategizing to develop more proactive health services, access to their customers’ health data is necessitated, making health data an integral part of their supply chain. Definitions of the 4 SET constructs are summarized in Table 1. Columns 1 and 2 of Table 1 show the SET construct as well as a summary of the definition of the respective construct based on the study by Wu et al [38]. Furthermore, they are contextualized to elucidate how the framework was used in the analysis of the survey data and the delineation of the customer expectation. Column 3 describes how the SET constructs were present in our data. Column 4 gives an example of how the expectations identified in the empirical data were interpreted and can be *read* in the context of the SET constructs.

**Table 1:** Summary of the social exchange theory framework definitions, application for data analysis, and expectation delineation.

Social exchange theory construct	Summarized definition [38]	Meaning in data analysis	Explanation of expectation
Commitment	Commitment is the inclination to achieve a shared purpose with the ongoing expectation to conditionally maintain the relationship	Activities for health data processing that motivate the formation and stabilization of the relationship between Omega and its customers	Customers expect Omega to commit to certain conditions when sharing their health data
Power	Power is the expectation that the partner with control over the desired resources influences the behavior of the other dependent partner	How Omega uses health data as part of its decision-making process for their customers regarding health-related outcomes	Customers expect Omega to use its power to act in either a positive or a negative manner when health data are being shared
Reciprocity	Reciprocity is the expectation of mutually beneficial outcomes between partners	Entering or perpetuating a relationship that offers perceived advantages for both Omega and its customers when using health data	Customers expect Omega to reciprocate with some form of a benefit in exchange for sharing their health data
Trust	Trust is the ability to establish equal confidence between partners with the expectation that the partners will act in each other’s best interest	Indications of what behaviors would increase the customers’ confidence in Omega when providing Omega with their health data	To be able to trust Omega with shared data, customers expect Omega to behave in a certain manner

### Ethical Considerations

No ethical declaration was required as the survey data used by the researchers were anonymous, no customer under the age of 18 years was asked to participate, and no identifying personal data were requested, only attitudes and opinions. Full disclosure text on the purpose of the study, the affiliation with a Horizon 2020 project, and the expected length of time to take the survey (between 5 and 10 min) were made available to all customers during the commencement of the survey in 3 languages.

## Results

### Customer Characteristics

Of the 5000 customers who were sent the survey, 452 surveys (response rate: 9.0%) were completed, and all were determined eligible. Table 2 presents the customer characteristics. The results of the customer characteristics were compared with internal demographic models at Omega from January 2018, and the survey sample was confirmed to be representative of Omega’s customer base. In addition to customer characteristics are the 3 result categories that did not fit within the SET framework, as the answers in the survey were skipped, customers reported having no expectations, or customers were unwilling to enter the exchange relationship. First, 10.6% (48/452) of customers intentionally skipped the open-text question in the survey, which was mandatory. This was typically denoted with repeated punctuation such as “?????” Second, 12.6% (57 of 452 customers) of the total responses included some variation of “I can’t really say.” These responses indicated that the customers had no expectations when sharing health data. Finally, 9.7% (44/452) of customers indicated an unwillingness to share health data, thus abstaining from the exchange relationship. Customers similarly stated, “I wouldn’t share my health data” (Customer 254). Their unwillingness to share their data was grounded in the opinion that Omega had no business with their health data or that their health data were private. Furthermore, customers emphasized that their health data were not for sale and that there is no motivator or incentive that would influence their willingness to share health data.

We identified several expectations that customers had in relation to sharing their health data with Omega. Table 3 summarizes the expectations resulting from our analysis, displaying the SET construct (column 1 and first-order), the larger themes that we grouped the expectations into (second-order), the actual expectations (third-order), and the percentages of answers in which this expectation was expressed (column 2). It is possible for an answer to be coded in more than one of the constructs as the responses typically included more than one theme. For example, a passage may contain a desire for something in return for sharing health data and a fear of not receiving compensation for it. This means that the percentages provided in the results (Table 3) are not summative. Customers’ responses that expressed an unwillingness to share health data (44/452, 9.7%) and thus an unwillingness to enter the social exchange overall could not be classified with SET and are not included in Table 3. Furthermore, customers’ responses that expressed no expectations when sharing health data (57/452, 12.6%) were not included in Table 3.

Next, we describe our findings in detail.

**Table 2:** Customer characteristics representing the number of customers (N=452) and their reported gender, age, and highest level of education. Customers disengaged from the exchange relationship are also included.

Characteristics		Customers, n (%)
**Gender**		
	Female	227 (50.2)
	Male	224 (49.6)
	Other	1 (0.2)
**Age range (years)**		
	18-24	11 (2.4)
	25-34	43 (9.5)
	35-44	87 (19.2)
	45-54	99 (21.9)
	55-64	104 (23.0)
	65-74	86 (19.0)
	≥75	22 (4.9)
**Highest level of education**		
	Primary or comprehensive school	32 (7.1)
	High school or vocational school	145 (32.1)
	Some college credit, no degree	30 (6.6)
	Bachelor’s degree	146 (32.3)
	Master’s degree	98 (21.7)
	Doctoral degree	1 (0.2)
**Customers disengaged from the exchange relationship**		
	Skipped survey responses	48 (10.6)
	No expectations when sharing	57 (12.6)
	Unwillingness to share	44 (9.7)
	Total customers	149 (33.0)

**Table 3:** Expectations of insurance customers for sharing health data (N=452).

Social exchange theory construct, theme, expectation			Customers, n (%)
**Commitment**			
	**Requirements for data treatment**		
		Access and control	7 (1.5)
		Security	16 (3.5)
		Privacy	10 (2.2)
		Use	31 (6.9)
		Total customers	64 (14.2)
**Power**			
	**Negative consequences**		
		Policy	4 (0.9)
		Profiling	18 (4.0)
		Total customers	22 (4.9)
**Reciprocity**			
	**Compensation**		
		Discounts	128 (28.3)
		Tangible goods	7 (1.5)
		Total customers	135 (29.9)
	**Benefits**		
		Customer experience	14 (3.1)
		General advantages	52 (11.5)
		Personalization	63 (13.9)
		Total customers	129 (28.5)
**Trust**			
	**Empathy**		
		Compassion	8 (1.8)
		Confidence	13 (2.9)
		Transparency	13 (2.9)
		Total customers	34 (7.5)

### Commitment

Commitment is the inclination to maintain the exchange relationship on certain conditions. Customers expected Omega to meet certain requirements for treating their health data. These requirements were access and control, security, privacy, and use.

#### The Requirement of Access and Control

Customers expected Omega to facilitate access and control to their health data. A total of 1.5% (7/452) of customers conveyed that they require access to their health data in some form and that access to their health data should be strictly regulated and only available to pertinent persons. Customers also valued the presence of accountable actions when access to health data occurs by tracking and logging why the data were accessed and who accessed them; this data log should also be made available to the customers upon request: “I would see who accesses my information” (Customer 396). Having access control to health data is also expected by customers to manage their health data to perform tasks such as correcting any misinformation, managing what information is shared, and deleting information if desired.

#### The Requirement of Security

Customers expected Omega to act as a data guardian to ensure the security of their health data. Overall, 3.5% (16/452) of the customers specifically mentioned that they would expect Omega to store and secure their data against external attacks. As part of the requirements for data treatment, customers expect technical and legal measures to be in place for storing, processing, and controlling their health data: “Taking care of information security” (Customer 234).

#### The Requirement of Privacy

A total of 2.2% (10/452) of customers expected Omega to act with a high degree of discretion, much like the belief in those who swear by the Hippocratic oath, that is, only those who are authorized to engage with the data will do so and will also conduct themselves with the level of confidentiality seen in health care professionals. This is illustrated by the following quote: “Similar restrictions and confidentiality obligations in relation to accessing the data, that exists in the healthcare sector...” (Customer 195).

#### The Requirement of Use

Customers expected Omega to provide details about the use of their health data in the insurance organization. Overall, 6.9% (31/452) of customers expressed that knowing the use or purpose of the data is an important condition for sharing their health data. The sentiment of being informed was echoed repeatedly: “Complete information about what the data will be used for” (Customer 102). This signifies that there is a current lack of or no understanding of what Omega would use health data for. Furthermore, 7 of the 31 customers reporting on their data use expectations explicitly were concerned about the *dark side* of use, that is, they expected assurances that their data were not going to be sold or used by external parties and that the terms of use would not be altered without their consent: “A hundred per cent certainty that the information will only be used for means that have been agreed on together, and Omega won’t unilaterally expand the [means of] use” (Customer 201).

### Power

Power is the level of influence one partner has by controlling access to desired resources. Omega has the capacity to make positive or negative decisions that affect the health and well-being of customers and to deny or limit financial support. Customers expected that sharing their health data would lead to Omega abusing their power in the form of misleading policy and health profiling.

#### The Negative Consequence of Policy

Customers expected Omega to purposefully make policy documentation difficult to understand in layman’s terms. A total of 0.9% (4/452) of customers expected that Omega would intentionally mislead their customers through unclear policy practices: “You can’t expect anything from the kind of insurance company that cheats their customers with nonexistent insurance policies” (Customer 62). All 4 customers were fearful that Omega would leverage their power through fine print in their policies to avoid compensating their customers in the case of an accident or illness.

#### The Negative Consequence of Profiling

Some customers expected Omega to use the health data they shared against them by profiling their health. However, a surprisingly small number of responses had negative expectations when sharing their health data with Omega. Overall, 18 of 452 customers (response rate: 4.0%) thought that sharing their health data would ultimately lead to Omega using these data to charge the customers more money, reduce compensation, and utilize the health data to exert their power over the customer: “I wouldn’t be ready to share my data because I believe that the issue would always be flipped around to be the customer’s fault by using their health data, if compensation was required” (Customer 127). No positively oriented responses were made by customers.

### Reciprocity

Reciprocity is the facilitation of mutually beneficial outcomes for partners in an exchange. Reciprocity is connected to the perceived values of each party. Customers provided extensive and detailed expectations for getting something in exchange for sharing their health data, which indicates that they perceive their health data to be valuable enough to merit something in return. Reciprocity is split into 2 themes: compensation, which is finance-related rewards for sharing health data, and benefits, which provide the customer with an advantage they deem worthy.

#### Discounts as Compensation

Customers expected discounts in exchange for sharing their health data, placing most of their perceived value on decreasing the overall insurance costs. A total of 128 of 452 customers (response rate: 28.3%) expected a form of financial compensation for sharing their health data through discounted insurance payments or services: “Lower insurance payments” (Customer 367). Despite the total number of responses falling under this theme (135/452, 29.9%), there is not much variation between financial compensation expectations. Customers generally indicated that shared health data provide value to Omega and expected to pay less in return. However, some of the customers emphasized that this discount should be substantial, significant, or in the form of “a whopping discount on insurance policies” (Customer 35).

#### Tangible Goods as Compensation

A total of 1.5% (7/452) of customers expected compensation in the form of tangible goods. Furthermore, 3 customers specifically requested a fitness device for monitoring purposes: “A device with which activity, exercise, and heartbeat is monitored” (Customer 382). The remaining 4 customers expected outright money in exchange as their privacy would be decreased or because Omega would be making additional profits: “Money and a lot of it...” (Customer 369).

#### Benefits of a Better Customer Experience

A total of 3.1% (14/452) of customers expected Omega to be able to provide a better customer experience if it has additional information (in the form of their health data). The majority of answers were vague as to what better customer services meant and how it would be implemented. However, some did specify what more meaningful services meant for them. Emphasized among them was the desire for standardization when it came to decisions about compensation for insurance claims, so that it would not matter which insurance clerk made the decision as it would always be the same decision, as highlighted in the following quote: “I would expect equality in processing, so that decisions wouldn’t change based on who handles them” (Customer 36). This was also coupled with the desire to have a relationship with an employee to create a smoother and more familiar customer experience.

#### Benefits in the Form of General Advantages

Customers expected general advantages when sharing health data with Omega in a variety of forms. A total of 11.5% (52 of 452 responses) of the open-text answers alluded to an advantage in a vague form. This was typically characterized by a demand for “Perks” (Customer 153) or “Bonuses!!” (Customer 317). The advantage could also take the form of services where customers repeated the expectations for “good service” (Customers 133, 181, 259, and 386). However, there was no indication of what would specifically improve the services offered by Omega for these customers. Furthermore, 4 customers suggested that loyalty benefits would encourage the sharing of their health data with Omega: “The benefits for regular customers should be significantly better” (Customer 99).

#### Benefits of Personalization

One of the largest saturated results from this survey was that of personalized health services. A total of 13.9% (63/452) of customers expected some form of tailored help with their health in the form of personalized digital health services. The majority of these customers desired help with living a healthier lifestyle or improving their overall health through these customized services, as stated by a customer, “A proactive take on what [kind of things] one should watch out for” (Customer 21). However, many customers took it a step beyond proactive digital health services by also requesting that their insurance plans reflect this personalization as well, such as “[personalized] services and insurance policies ‘designed’ for me” (Customer 69). Customers also expected that having good health or living a healthy lifestyle would reduce their risk category. Supplemented by health data as proof of their health status and shared with Omega, the overall cost of their health services should decrease as they represent a decreased risk for expense.

### Trust

Trust is the ability to establish confidence between partners. Customers expected Omega to show empathy by being compassionate, confident, and transparent with them.

#### Empathy Through Compassion

Customers expected compassion from Omega as they believed that sharing health data makes the relationship more human. In total, 1.8% (8/452) of customers described scenarios where they wished Omega would be compassionate or more understanding of their customers. Echoed in the open-text answers are expectations that during the turmoil of illness, Omega would strive to ensure that the insurance process is a source of support, not a burden. The following quote illustrates the expectations for emotional support in difficult life situations: “When falling seriously ill, one would get treatment, and wouldn’t need to wrestle with the insurance company about finances” (Customer 314).

#### Empathy Through Confidence

Customers expected Omega to show confidence in its customers. To share health data, customers will be giving Omega the authority to process and control their data. Overall, 2.9% (13/452) of customers expected that sharing health data is aligned with absolute trust between themselves and Omega and that trust should be mutual. Therefore, Omega needs to conduct itself in a manner the customers deem trustworthy because by sharing health data, customers are establishing themselves as trustworthy. This was highlighted in the comments as “unconditional trust” (Customer 27). No specific mechanism is mentioned for what actions would enable mutual trust in practice.

#### Empathy Through Transparency

Customers expected transparency from Omega about its actions and intentions with health data. Similar to the conditions for data treatment (use), 2.9% (13/452) of customers wanted Omega to be more transparent about use. This was not just specific to health data but rather the organization’s processes in general. They expected Omega to provide fair and open treatment to its customers as well as provide clear insurance policies that convey simple and meaningful information: “Fair, transparent, and egalitarian treatment” (Customer 284). Only 2 customers (Customers 195 and 431) suggested how Omega might enact this transparency, both suggesting external validation: “Transparency in the handling. Some outside body to evaluate the insurance company’s compensation verdicts” (Customer 431). This indicated a certain level of distrust in Omega, despite Customer 431 purchasing multiple insurance services from Omega.

## Discussion

### Principal Findings

In this research, we asked what insurance customers would expect in return for sharing their health data. Our contribution was three-fold. First, we identified concrete customer expectations. Second, we determined the perceived values that are intrinsic to health data. Third, we explored the conditions that aid in incentivizing health data sharing in the relationship between an insurance organization and its customer. Our findings contribute to research on health data sharing to aid in the design and development of proactive digital health services [12,13]. Next, we discuss each of our contributions.

First, with regard to the expectations of customers, our findings showed that the majority of customers saw their health data to be valuable and thus wanted something in exchange. We classified these expectations with the help of SET and identified that customers expect the organization to be committed to fulfill certain requirements for data treatment (eg, access and control of health data), to provide the customer with some form of compensation or benefits (eg, personalization of health services) in reciprocity, and to show empathy toward the customers to increase the customers’ trust (eg, by being transparent about the use of health data). The majority of Omega’s customers expected reciprocal advantages in exchange for sharing their health data, hinging on the contingency that they would receive personalized digital health services that would help to prevent illness in their lifetime. This finding is congruent with the quantitative data of this survey presented in another paper [18]. Customers also expect digital health services to provide personalized and proactive interventions, a trade-off between the concerns of information privacy and the perceived value of digital health services [39]. A surprisingly small number of customers (22/452, 4.9%) expected a negative outcome when sharing their health data either through profiling or using policy to assuage insurance claims, a consequence of Omega’s perceived power.

Second, our results had some indication of the perceived value that is intrinsic to health data. Our findings showed great diversity in the value that customers ascribe to their health data. On the one end of the continuum are those who said that they want nothing in return for sharing their health data, thus ascribing a very low value to health data. On the other end are those who expressed that they would not share their health data with the company under any circumstances, thus ascribing a very high value to the data. In between these extremes, almost a third of the total expectations detailed a form of benefit or compensation as a reciprocal condition in the exchange relationship. Some customers, for example, expected money or tangible goods for sharing their health data. Interestingly, this gives health data a specific value, such as the price of a fitness tracker. For example, the approximate price of the base Fitbit Flex available is US $99, whereas the Fitbit Charge HR is slightly more expensive at US $149; the Apple watch varies greatly in price starting from approximately US $349 [40]. Perhaps the most significant finding was that health data are perceived to enable a shift of power from the insurance company to the customer. The understanding that the insurance company has power over the customer was present in several forms, including how insurance companies make decisions about compensating customer claims. This decision is made based on the information that is available to the insurance company through the claim process and is supported by Finnish legislation [19]. As power and data are intrinsically linked, data can be understood to be a form of currency, and customers understand that sharing their health data requires an exchange of power for this currency. To balance the inequality of power, conditions such as data treatment help shift the asymmetry of power away from the dominance of the insurance industry and more toward the customers. However, this requires strategic movements by the insurance organization to ascribe the customers’ value in business models by incentivizing data sharing [9]. As made evident in our analysis, the customer (unconsciously or consciously) is willing to share health data if certain conditions are met to equalize the power in the relationship.

Insurance organizations, similar to most health care stakeholders, require access to health data [9]. As our third contribution, and based on the identified expectations, we were able to ascertain certain conditions that should be met by the insurance organization to incentivize health data sharing and correct negative perceptions held by the customers. Our results showed a certain lack of trust as some customers are skeptical about the trustworthiness of specific processes and policies in insurance contexts. Customers assume that complicated legal jargon and fine print writing in contract agreements will impede them from receiving health insurance coverage. Complex insurance policies act as barriers to a customer’s access to health coverage, such as medication or surgery, potentially harming rather than helping customers [41]. Through transparent actions and by establishing a culture of transparency, customers could better understand how their health data will be used [42]. Increased transparency should also focus on decreasing the fear of health data being used to prevent insurance coverage provision or to increase insurance costs for individuals based on their health data.

Previous research shows that patients’ access to their Web-based health data empowers them to make more informed decisions and supports a more proactive role in their health [43-45]. Primarily because of legal actions, such as the GDPR, and empowering movements, such as MyData [7,10], individuals now have more digital power than ever before and control how their health data are shared. Our findings indicated that customers are also aware of their rights, as 14.2% (64/452) of customers expressed certain data treatment requirements. Thus, the organization should make explicit how it will treat the customers’ health data, who will have access to the data, how the data will be used, and what measures will be taken to protect the data from unwanted access. In addition, some customers either had no expectations when sharing health data (57/452, 12.6%) or preferred to not engage in an exchange relationship with Omega (44/452, 9.7%). This is interesting when comparing our findings with a Canadian primary care practice context, which reported a much higher degree of unwillingness to share data with private insurance companies (79% of physicians and 67% of patients) [20]. However, passive exchange partners can still form relationships without engaging in health data sharing beyond what is necessary, such as initial screening for health insurance. For Omega and the health data economy, this suggests that some individuals will be passive or even indifferent participants, some of whom would need additional stimulation or education to meaningfully be incentivized to engage in digital health solutions [3,12,46].

### Limitations

There are some limitations to this study that are worth mentioning. First, a major criticism of SET is that it is most useful in describing post hoc patterns but has limited utility in pinpointing specific a priori predictions [47]. However, we used the SET framework as a way to develop an understanding of how customers perceive value in health data, providing conditions for insurance organizations to make future strategic movements for health data sharing, not predicting how the customer will act. Second, although our survey provided a rich source of data, the response rate could have been higher, given more time and resources to send follow-up fill-in reminders or prompts through other means. Third, despite our best efforts to verify that only the intended 5000 customers who fit our inclusion criteria (ie, an active customer, >18 years of age, and with an email address) participated in the survey, a relatively small number may not have been active customers. The final limitation, also related to our survey, is the response bias, as those who were more willing to fill in the survey may also be more willing to engage with Omega in other areas such as sharing health data, meaning that those who contributed represent a higher percentage of people who might wish to participate in sharing health data. However, as our survey results captured a diverse range of responses, in our view, Omega’s customers are well represented.

### Conclusions and Future Research

Data sharing is the foundation on which the health data economy can be formed to be mutually beneficial for all health care stakeholders [3]. Our survey found that the majority of customers in an insurance organization are open to exchanging their health data under certain conditions. However, it is apparent that no single offering or exchange for customers can apply as a *one-size-fits-all* solution. Personalization of research streams should aim to cover the scope of need of all customers in digital health services to aid in design and development.

In the case of reciprocal benefits to customers, there is the potential to perpetuate the exchange of health data under the right conditions. The ongoing collection and sharing of data from activity monitors provided by insurance organizations positively affect willingness to share data when customers perceive the benefits of sharing to have a positive impact on their health and wellness [48]. Organizational research should focus on the development of trust between the organization and its customers to improve proactive digital health services so that those services would provide more value to the customers. The power possessed by insurance organizations highlights the negative side of data sharing, and acts of transparency to increase trust could help alleviate this negative valence. Fortuitously, Omega is aware that transparency is a concern [19]. Future research should aim to understand transparent actions and how they can be implemented in a manner that shifts the balance of power for mutually beneficial outcomes.

## Abbreviations

GDPR: General Data Protection Regulation
SET: social exchange theory
